# Antiembolic stockings and pneumatic compression devices in a medical-surgical thromboprophylaxis trial

**DOI:** 10.1186/cc9441

**Published:** 2011-03-11

**Authors:** N Zytaruk, D Heels-Ansdell, S Vallance, J Marshall, Y Skrobik, DJ Cooper, S Finfer, I Seppelt, M Ostermann, I Qushmaq, M Alsultan, Y Arabi, J Alhashemi, M Al-Hazmi, A Alzem, N Shaikh, Y Mandourah, DJ Cook

**Affiliations:** 1 McMaster University, Hamilton, Canada; 2 Alfred Hospital, Melbourne, Australia; 3 St Michael's Hospital, Toronto, Canada; 4 Maisonneuve Rosemont, Montreal, Canada; 5 The George Institute, Sydney, Australia; 6 Nepean Hospital, Sydney, Australia; 7 Guy's & St Thomas' Hospital, London, UK; 8 King Faisal Hospital, Jeddah, Saudi Arabia; 9 King Abdulaziz Hospital, Ryiadh, Saudi Arabia; 10 King Abdulaziz Hospital, Jeddah, Saudi Arabia; 11 King Fahad Hospital, Ryiadh, Saudi Arabia; 12 Riyadh Military Hospital, Riyadh, Saudi Arabia

## Introduction

A recent randomized trial (CLOTS-1) has called into question the utility of antiembolic stockings (AESs); another trial (CLOTS-2) suggested harm with below-knee compared with above-knee AESs. AESs and pneumatic compression devices (PCD)s could represent important co-interventions in a heparin thromboprophylaxis trial if exposure was lengthy and frequent. Our objective was to document the use of AESs and PCDs applied per protocol and by protocol violation in a trial comparing UFH versus LMWH in medical-surgical ICU patients (NCT00182143).

## Methods

A total of 3,659 patients were recruited internationally. The blinded study drug was administered daily in the ICU. Mechanical prophylaxis was only protocolized for use if anticoagulant prophylaxis was contraindicated (major bleeding, high risk for major bleeding, or suspected or proven heparin-associated thrombocytopenia). Research coordinators prospectively documented daily exposure to study drugs and mechanical prophylaxis.

## Results

A total of 3,659 patients were enrolled for a median (IQR) ICU stay of 9 (5, 16) days. AESs were used per protocol in 17.1% of patients for 1 (1, 1) day; 14.1% of the patients had knee-length stockings. AESs used in violation of the protocol occurred in only 2.6% of patients (1.9% of the patients had knee-length stockings), for which the duration of exposure was 1.5 (1, 4) days. PCDs were used per protocol in 11.1% of patients for 1 (1, 3) days, and in 1.8% of patients for 2 (1, 3) days in violation of protocol.

## Conclusions

In keeping with uncertain effectiveness of mechanical thromboprophylaxis, and emerging evidence about harm with knee-length stockings, the co-intervention of mechanical thromboprophylaxis on the results of the PROTECT testing anticoagulant thromboprophylaxis trial will be minimal. AES and PCD use was brief, and largely reserved for days when heparin was contraindicated, as per clinical practice.
